# Phenolic Profile, EPR Determination, and Antiproliferative Activity against Human Cancer Cell Lines of *Anthyllis vulneraria* Extracts

**DOI:** 10.3390/molecules27217495

**Published:** 2022-11-03

**Authors:** Manel Ouerfelli, Isidoro Metón, Idoia Codina-Torrella, María Pilar Almajano

**Affiliations:** 1 Chemical Engineering Department, Escola Tècnica Superior d’Enginyeria Industrial de Barcelona (ETSEIB), Universitat Politècnica de Catalunya, Av. Diagonal 647, 08028 Barcelona, Spain; 2 Biology Department, Faculty of Sciences of Tunis, University of Tunis El Manar, Tunis 2092, Tunisia; 3 Biochemistry and Physiology Departament, Facultat de Farmàcia i Ciències de l’Alimentació, Universitat de Barcelona, Joan XXIII 27–31, 08028 Barcelona, Spain; 4 Agri-Food Engineering and Biotechnology Department, Escola d’Enginyeria Agroalimentària i de Biosistemes de Bacelona (EEABB), Universitat Politècnica de Catalunya, Esteve Terrades 8, 08860 Castelldefels, Spain

**Keywords:** *Anthyllis vulneraria*, HPLC-MS, EPR, methoxy radicals, antiproliferative activity, human-derived cancer cells

## Abstract

In the current work, the leaf and flower extracts of *Anthyllis vulneraria* were evaluated for their chemical characterization using HPLC-MS and for their radical scavenging capacity toward methoxy radicals produced by a Fenton-type reaction using an electron paramagnetic resonance (EPR) spectroscopy assay. The in vitro antiproliferative activity of these extracts against several human-derived cancer cells (breast: MCF-7; cervical: HeLa; hepatocellular: HepG2) was also evaluated. The results showed that the *Anthyllis vulneraria* leaf extract was characterized by 17 different phenolic compounds, among which phenolic acids were the most abundant, while its flower extract exhibited higher contents of flavonoids. Furthermore, *Anthyllis vulneraria* extracts demonstrated a potent radical scavenging activity against methoxy radicals. Both extracts also significantly reduced the viability of the different cancer cell lines. The results of the current study suggested that *Anthyllis vulneraria* extracts are a promising source of antioxidant compounds with health benefits and pointed to their potential use for treating cancer and developing novel therapeutic agents.

## 1. Introduction

Cancer remains a leading cause of death worldwide, and recent research confirms that the number of people with cancer will continue to grow for at least the next two decades [1]. This group of diseases starts when abnormal cells grow uncontrollably and invade different parts of the body [2]. Exposure to risk factors plays an important role in the biology and burden of many cancer types [3], and most cancers can be prevented by modifying key risk factors or implementing prevention strategies [4].

Living beings are permanently exposed to various forms of stress and aggression, which cause temporary disturbances in organism functioning [5]. Faced with these occasional situations, organisms’ response includes rapid adaptation for efficient protection, although normal adaptive mechanisms could not be sufficient and lead to the induction of oxidative stress [6]. This oxidative stress becomes abnormal when the cells of biological organisms are overwhelmed by reactive oxygen species (ROS) [7], which are toxic agents that provoke several health dysfunctions and are considered inflammatory mediators involved in various chronic diseases [8], such as certain cancers [9]. Recent research has demonstrated that ROS can specifically activate certain signaling pathways and thus contribute to tumor development through the regulation of cell proliferation, angiogenesis, and metastasis [10].

In spite of the domination of synthetic human-made chemicals to produce drugs and treat chronic diseases, medicinal plants are still the main source of bioactive compounds used in the pharmaceutical industry to develop new products [11]. The evaluation of the therapeutic value of these bioactive molecules is the subject of several studies, which are focused on the identification of the main active elements of the extracts of these plants for their potential biological activity and medicinal value [12,13].

The industrial interest in plant extracts is partly due to recent studies about the antioxidant activity and physiological value properties of phenolic compounds (anti-inflammatory, anticarcinogenic, cardioprotective, and vasodilator activities, among others, which are mainly attributed to the important role of these components in the oxidative destruction processes (free radical neutralization, oxygen scavenging or peroxide decomposition)) [14]. In fact, recent epidemiological studies have demonstrated that high consumption of antioxidant-rich fruits and vegetables reduces the risk of many cancers, which confirms that antioxidants could be effective agents for the prevention and cure of this disease [15].

*Anthyllis vulneraria* L. (*A. vulneraria*) is a medicinal plant that belongs to the Fabaceae family and is commonly cultivated in different Mediterranean mountainous regions. The extracts of this plant have been traditionally used for treating different diseases (wounds, burns, skin and stomach inflammation, eczemas, etc.), and it has also been recognized as a cough suppressant or a depurative and diuretic plant [16]. The leaves and flowers of *A. vulneraria* are rich in phenolic compounds (tannins, saponins, carotenoids, or flavonoids, among others), so the extracts obtained from these vegetative parts present an important antioxidant activity [16]. In our previous study [17], it was reported that the total polyphenol content of the leaf and flower extracts of *A. vulneraria* exhibited ~83 and ~134 mg gallic acid equivalent/g dry weight, respectively, and their total flavonoid content ranged between ~20 and 34 mg quercetin equivalent/g dry weight, respectively. These studies revealed that the extracts from these tissues presented a strong antioxidant activity (the best antioxidant activity corresponded to flower extracts, with higher values than those observed in leaf extracts (74, 67, 73, and 94% determined by ferric reducing antioxidant power (FRAP), oxygen radical absorbance capacity (ORAC), Trolox equivalent antioxidant capacity (TEAC), and DPPH assays, respectively), and they were also effective in retarding lipid oxidation reactions in oil-in-water emulsions. Similar results have been reported by other authors in the literature, such as Godevac et al. [18], who determined the antioxidant activity of *A. vulneraria* growing in Serbia and Montenegro, and Tusevski et al. [19], who studied the phenolic production and antioxidant properties of *A. vulneraria* collected from Galichitsa mountain in Republic of Macedonia, although the characteristics of the extraction method and the biological stage of the plant highly affect the distribution of these bioactive components on plants’ vegetative parts and also determine the composition of these extracts.

Taking these findings into account, the present study was conducted to evaluate the antiradical and antiproliferative activities of the leaf and flower extracts from *A. vulneraria* against different cancer cell lines (breast: MCF-7; cervical: HeLa; hepatocellular: HepG2). To the best of our knowledge, no previous studies were conducted to evaluate *A. vulneraria* CH_3_O^−^ radical scavenging capacity using spin trap and EPR techniques. On the other hand, 17 compounds, mainly phenolic acids, responsible for these activities have also been identified. In terms of all the above aspects, the current research will complement the available information.

## 2. Results and Discussion

### 2.1. Phenolic Profile of A. vulneraria Extracts

Table 1 and Table 2 show the phenolic compounds identified in the leaf and flower extracts of *A. vulneraria*, respectively.

In the leaf extract (Table 1), 17 phenolic compounds were identified, which corresponded to 11 different phenolic acids, 1 flavonoid, and 5 other types of polyphenols. By comparison, 15 compounds were identified in the flower extract, which included 8 phenolic acids and 7 flavonoids. Concerning the phenolic acids in the leaf extracts, nine of them were identified using a negative mode of ionization: chlorogenic acid (L2, with [M − H]—at *m*/*z* 353.0878 and 353.0880), caffeic acid (L4, with [M − H]—at *m*/*z* 179.0345 and 179.0350), 2, 3-dihydroxybenzoic acid (L6, with [M − H]—at *m*/*z* 153.0193 and 153.0203), p-coumaric acid (L7, with [M − H]—at *m*/*z* 163.0401 and 163.0393), ferulic acid (L9, with [M − H]—at *m*/*z* 193.0506 and 193.0502), sinapinic acid (L10, with [M − H]—at *m*/*z* 223.0612 and 223.06603), 4-hydroxy-2-phenylacetic acid (L11, with [M − H]—at *m*/*z* 151.0400 and 151.0408), and 2-hydroxybenzoic acid (L13, with [M − H]—at *m*/*z* 137.0244 and 137.0249). Only two phenolic acids were identified using a positive mode of ionization, which corresponded to cinnamic and 4-hydroxyphenylpropionic acids (L12, with [M + H] + at *m*/*z* 149.0597 and 149.0587 and 16, with [M + H] + at *m*/*z* 165.0557 and 165.0509, respectively).

Concerning the flavonoids, kaempferol-3-O-rutinoside was identified with [M − H]—(L17, at *m*/*z* 285.0404 and 285.0404), whereas the rest of the compounds were represented as other polyphenols, with [M − H] − and included 4-hydroxybenzaldehyde (L3, with [M − H]—at *m*/*z* 121.0295 and 121.0306), 3, 4-dihydroxyphenylglycol (L5, characterized at *m*/*z* 169.0506 and 169.0503) and p-anisaldehyde (L8, assigned as at *m*/*z* 135.0451 and 135.0456). As shown in Table 1, other polyphenols with [M + H] + corresponded to pyrogallol (L1, at *m*/*z* 127.0390 and 127.0391) and coumarin (L14, at *m*/*z* 147.0441 and 147.0429).

Table 2 shows the phenolic compounds identified and quantified in the *A. vulneraria* flower extract. All phenolic acids were identified using a negative mode of ionization: chlorogenic acid (F1, at *m*/*z* 353.0878 and 353.0880), caffeic acid (F2, at *m*/*z* 179.0345 and 179.0350), syringic acid (F3, at *m*/*z* 198.05282 and 197.0453), p-coumaric acid (F5, at *m*/*z* 163.0401 and 163.0393), ferulic acid (F6, at *m*/*z* 163.0401 and 163.0393), sinapinic acid (F7, at *m*/*z* 223.0612 and 223.0603), 2, 3-dihydroxybenzoic acid (F8, at *m*/*z* 153.0193 and 153.0203), and p-coumaroyl tartaric acid (F12, at *m*/*z* 295.0459 and 295.0468).

As shown in Table 2, among the seven flavonoids detected, only two were identified using a positive mode of ionization: quercetin (F9, at *m*/*z* equal to 303.05 and 303.0487) and myricetin (F10, at *m*/*z* of 319.0449 and 319.0427), while the others were characterized using a negative mode of ionization: epicatechin (F4, at *m*/*z* 289.0717 and 289.0717), quercetin (F11, at *m*/*z* 301.0354 and 301.0375), delphinidin 3-O sambubioside (F13, at *m*/*z* 596.1383 and 596.1363) rutin (F14, at *m*/*z* 609.1525 and 609.1509), and kaempferol –3–O–rutinoside (F15, at *m*/*z* 285.0404 and 285.0404).

The results obtained in the current study exhibited that the *A. vulneraria* leaf extract was characterized as an abundant source of phenolic acids (Table 1), with its contents ranging between 49.58 (p-coumaroyl tartaric acid) and 7985.14 µg/g DW. As shown in Table 1, the most abundant compounds of this fraction corresponded to chlorogenic, caffeic, 2,3-dihydroxybenzoic, p-coumaric, ferulic, sinapinic, 4-hydroxy-2-phenylacetic, and cinnamic acids. Several studies have reported that some of these acids are related to different medical properties: caffeic acid, coumaric acid, and ferulic acid have antiproliferative and apoptotic effects on tumor cells, while sinapic acid shows a strong effect in inflammation diseases [27]. Monteiro Espíndola et al. [28] demonstrated the strong effect of caffeic acid against hepatocellular carcinoma. Additionally, Boo [29] reported the importance of coumaric acid as an active ingredient in cosmetics. As observed in Table 1, the only flavonoid detected in this fraction corresponded to kaempferol-3-O-rutinoside (6314.85 µg/g DW), whereas the other types of polyphenols represented the minor fraction. In contrast, the content of flavonoids in the flower extract was higher than the content detected in the leaf extract and varied between 64.24 (delphinidin 3–O sambubioside) and 6314.85 (kaempferol–3–O–rutinoside) µg/g DW (Table 2). As observed, the most abundant flavonoids corresponded to epicatechin, quercetin, myricetin, delphinidin 3-O sambubioside, rutin, and kaempferol-3-O-rutinoside. In this fraction, the content of phenolic acids ranged between ~41.77 and 6314.85 µg ferulic acid/g DW, among which sinapinic, p-coumaric, and caffeic acids corresponded to the most abundant compounds. These results corresponded to those previously observed by our group [17], in which the flower extract of *A. vulneraria* was found to contain a higher total polyphenol and flavonoid content (62% and 67%, respectively) than the leaf extract.

### 2.2. EPR Scavenging Activity of CH_3_O^–^ Radicals

**Figure 1:**
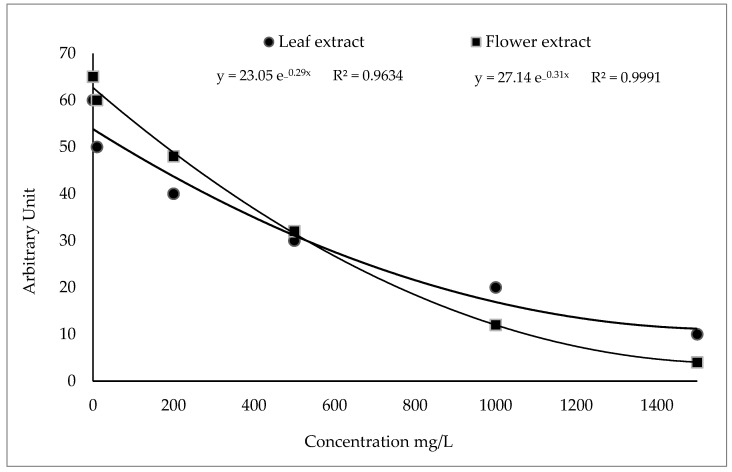
Decrease in the spectrum intensity of the spin adduct of CH_3_O^−^ with the increase in *A. vulneraria* leaf and flower concentrations.

The free radical scavenging activity of *A. vulneraria* leaf and flower extracts was determined by a competitive reaction in the presence of DMPO. Their antioxidant activity (leaf and flower extracts) was determined by a decrease in the intensity (DI) of the spectral bands of the adduct DMPO-OCH_3_ in the EPR signal (Figure 1).

**Figure 2:**
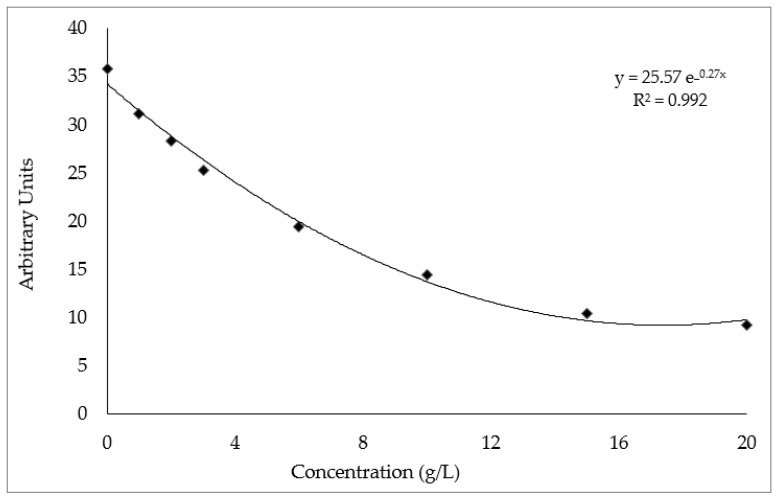
Decrease in the spectrum intensity of the spin adduct of CH_3_O^−^ with the increase in ferulic acid concentrations.

As shown in Figure 2, the best fitting function corresponded to the exponential curve obtained with the equation obtained with a ferulic acid solution. Both *A. vulneraria* extracts (leaf and flower) exhibited a negative relationship between their concentrations and the scavenging capacity of CH_3_O^−^ radicals, which could be correlated with the type and concentration of the phenolic compounds of each sample.

As shown in Figure 1, the arbitrary units of leaf and flower extracts decreased from 55 and 65 to 10 and 4, respectively, at concentrations ranging from 0 to 1500 mg ferulic acid/L MeOH.

Phenolic compounds are widely recognized as potent antioxidants and anticancer agents [30], mainly for their protective role against skin, cancer, or inflammation diseases. The antiradical potential of phenolic compounds depends on the pattern and degree of their hydroxylation [31]; the 4-oxo function on the rings A and C and the 3-hydroxyl group on the ring C in flavonoids form a good combination for a potent radical scavenging potential [32]. Moreover, the number and position of phenolic hydroxyls in phenolic acids and the methoxy and carboxylic acid groups are directly related to their antioxidant potential [33]. In the current study, the presence of a 3-hydroxyl structure in phenolic acids mainly detected in *A. vulneraria* extracts (ferulic acid, caffeic acid, p-coumaric acid, sinapic acid, p-hydroxybenzoic acid, and syringic acid, among others) enhances the antioxidant and antiradical activity of these samples. Previously, Ouerfelli et al. [17] observed the effectiveness of these extracts against lipid oxidation (the peroxide value and thiobarbituric acid reactive substances) of oil-in-water emulsions (10% purified sunflower oil, 1% Tween-20 and Milli-Q water) containing leaf and flower extracts, during their storage at 33 ± 1 °C for 30 days. The effect of powdered *A. vulneraria* leaf and flower extracts on the lipid oxidation of raw beef patties (11 days of storage, 4 °C) was also evaluated by these authors, who concluded that *A. vulneraria* extracts could be used as a potential natural additive in several industries (food, cosmetic, pharmaceutical, etc.).

### 2.3. Antiproliferative Activity (Viability-Reducing Activity) of A. vulneraria Extracts

**Figure 3:**
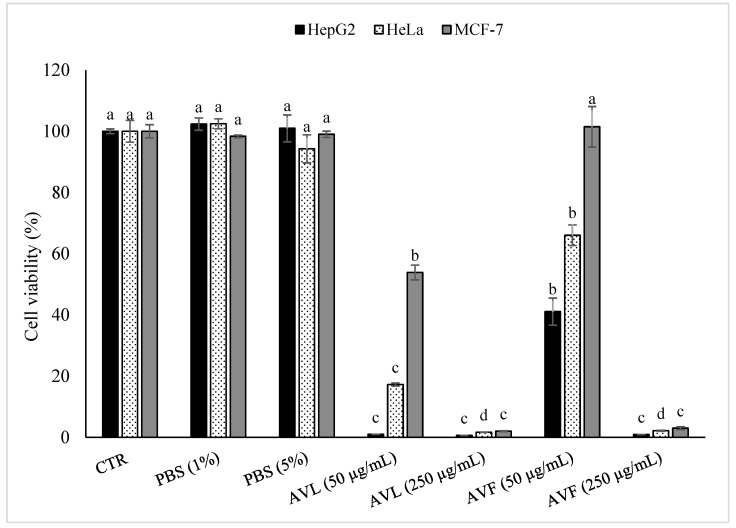
Effect of *A. vulneraria* leaf (AVL; 50 μg/mL and 250 μg/mL) and flower (AVF; 50 μg/mL and 250 μg/mL) extracts on cell viability assayed 48 h after treatment. CTR, non-treated cells; PBS, control cells incubated in the presence of solvent without *A. vulneraria* extracts. AVL and AVF extracts were dissolved in PBS and extracted PBS solutions were added at 1% or 5% in cell medium, which corresponded to a final concentration of 50 μg/mL and 250 μg/mL of extract, respectively. Error bars represent the standard deviation (*n* = 3). Lowercase letters indicate significant differences between treatments (CTR, PBS 1%, PBS 5%, AVL 50 µg/mL, AVL 250 µg/mL, AVF 50 µg/mL, AVF 250 µg/mL) for each cell line assayed (with α = 0.05).

As shown in Figure 3, no significant differences (with α = 0.05) were observed between the control and the samples treated with PBS (1% and 5%), but in contrast, the cancer cell viability significantly decreased (with α = 0.05) with the increase in the plant extract concentration (from 50 μg/mL to 250 μg/mL). Figure 3 shows that all the cancer cells treated with the *A. vulneraria* leaf extract (AVL) and *A. vulneraria* flower extract (AVF) at 250 μg/mL presented lower viability values than the cells treated with AVL and AVF at 50 μg/mL.

Concerning the type of extract, these results demonstrated that the leaf extracts (at 50 μg/mL and 250 μg/mL) exhibited better antiproliferative activity against the three-cancer cell lines tested (HepG2 > HeLa > MCF-7) than the flower extracts. The flower extracts at 50 μg/mL showed more effectiveness against hepatocellular carcinoma-derived cells (HepG2 > HeLa > MCF-7), while at 250 μg/mL, no significant differences (with α = 0.05) were observed among the evaluated samples. As shown in Figure 3, at the concentration of 50 μg/mL, the leaf extract reduced the viability of HepG2 cancer cells by 99.04%, whereas the HeLa and MCF–7 viability was reduced by only 82.75% and 46.13%, respectively. In contrast, the flower extract at 50 μg/mL was effective at reducing the viability of HepG2, HeLa, and MCF-7 cells by 58.93%, 33.97%, and 46.13%, respectively. At a higher concentration of these extracts (250 μg/mL), the results observed between both extracts (leaf and flower) were more similar. At this concentration, the leaf extract showed potent antiproliferative activity against all the cancer cells and reduced the viability of HepG2, HeLa, and MCF–7 cells by 99.37%, 98.35%, and 98.3%, respectively, while the flower extract showed slightly lower values and eliminated 99.11%, 97.85%, and 97% of HepG2, HeLa and MCF–7 cells, respectively.

In the current study, the highest antiproliferative activity observed in the *A. vulneraria* leaf extract could be associated with the presence of high levels of chlorogenic (5-fold higher in the leaf extract than in the flower extract) and ferulic acid (19-fold higher in the leaf extract than in the flower extract) of this sample. These two acids are recognized by their antitumor activity and also their potent capacity to inhibit metastasis in breast cancer cells by regulating the epithelial-to-mesenchymal transition [34]. Chlorogenic acid is also able to inhibit autophagy and induce cell cycle arrest in human cervical carcinoma cells [35]. Our findings suggest that the combined action of the phenolic compounds present in *A. vulneraria* extracts may determine the strong antiproliferative activity observed mainly in HepG2 cells and, to a lesser extent, HeLa cells. However, low levels of both leaf and flower extracts (50 μg/mL) had no significant effects on the viability of MCF-7 cells. Possibly, the metabolic features of MCF-7 cells vs. HepG2 and HeLa cells may explain the differential responses to *A. vulneraria* extracts. For instance, chlorogenic acid was reported to have a protective role against DNA damage through the increased formation of an amino acid derivative (S-adenosyl-L-homocysteine) in MCF-7 cells [36]. By contrast, HepG2 cell lines were the most affected (Figure 3). Given that HepG2 is an immortalized cell line derived from liver carcinoma cells, the higher sensibility of HepG2 cells to *A. vulneraria* extracts could be related to the central role of the liver in xenobiotic and drug metabolism, which can lead to the formation of highly toxic metabolites. Besides phenolic acids, flavonoids may also contribute to the potent antiproliferative effect of these extracts. For instance, a recent study conducted by Lim and Song [37] showed the inhibitory effects of delphinidin on the proliferation of ovarian cancer cells. Furthermore, myricetin is also reported to be able to suppress cancer cell invasion and metastasis, as well as to induce cell cycle arrest [38].

## 3. Materials and Methods

### 3.1. Reagents and Chemicals

DMEM (Eagle’s minimal essential medium), DMPO (5,5-dimethyl-1-pyrroline N-oxide), DMSO (dimethyl sulfoxide), EtOH (ethanol), ferulic acid, FeSO4 (iron(II) sulfate), formazan, formic acid, fetal bovine serum, MeOH (methanol), MTT (3-(4,5-dimethylthiazol-2-yl)-2,5-diphenyl tetrazolium bromide), PBS (phosphate-buffered saline), and SDS (sodium dodecyl sulfate), and sodium succinate were purchased from Sigma-Aldrich Química S.A (Madrid, Spain). Acetic acid, HPLC grade acetonitrile, and H_2_O_2_ (hydrogen peroxide) were acquired from Merck (Darmstadt, Germany).

### 3.2. Plant Samples

*A. vulneraria* was harvested from the mountains of the Zaghouan region, which is located in the north of the Tunisian ridge (latitude 36°, 24 min, 10 s north; longitude 10°, 08 min, 34 s east). Leaves and flowers were accurately separated from the rest of the plant and dried for ~2 weeks in the air (27 °C) until the achievement of constant weight. Then, the dried samples were ground using an electric blender (KRUPS F203, Barcelona, Spain). The homogeneous powder of each type of sample (leaves and flowers) was stored independently in amber glass bottles at room temperature until the performance of analyses.

### 3.3. Extract Preparation

The *A. vulneraria* extracts were prepared by dissolving 1 g of each dry powdered sample (leaves and flowers) in 20 mL of 50% EtOH (*v*/*v*) solution, for 24 h at 4 °C under stirring using a multiposition magnetic stirrer (Ovan, MM90E, Barcelona, Spain). The mixtures were then centrifuged (Orto Alresa Mod. Consul, Ajlvir, Madrid, Spain) at 1500× *g* for 10 min, and then the different supernatants were accurately separated and concentrated using a sample concentrator (Techne FSC496D sample concentrator, Madrid, Spain), under a jet of moderate nitrogen gas. The concentrated samples were lyophilized (for 2 days) using a freeze dryer (Unicryo MC2L, UniEquip Laborgerätebau and Vertr. GmbH, Munich, Germany).

The freeze-dried samples were dissolved in different solvents according to the following analyses: in 50% EtOH to perform HPLC-MS analyses, in deoxygenated pure MeOH to perform EPR analyses, and in PBS (5 mg/mL in all cases) to evaluate the antiproliferative activity toward cancer cell lines. Before proceeding with the different experiments, the extracts were filtered using sterile filters (0.22 µm diameter) (Teknokroma Analítica S.A. Barcelona, Spain). For all the parameters studied below, the samples were analyzed in triplicate.

### 3.4. HPLC-MS Instrumentation and Operating Conditions

The HPLC-MS analyses of the leaf and flower extracts were carried out using Agilent 1200 Series HPLC-MS equipment, consisting of an automatic sample injection system, two high-pressure isocratic pumps, a degasser, and a chromatographic oven. The different chemical components were separated using a C18 column (100 mm × 2.1 mm, 3.5 m, Zorbax Eclipse, Agilent, Madrid, Spain) connected to a C18 column (4 mm × 2 mm, Phenomenex, Torrance, CA, USA). The HPLC-MS conditions were set as follows: phase A: ultrapure water acidified with 0.11% formic acid; phase B: acetonitrile acidified with 0.11% formic acid, gradients: 0–2 min, 3% B; 25–27 min, 100% B; 28–38 min, 3% B, flow rate: 0.2 mL/min, injection volume: 10 µL, and column temperature: 30 °C.

Different commercial standards were subsequently used to identify the phenolic compounds detected by the HPLC-MS analysis. The tentative identification of the different components was confirmed by matching the retention time (RT) and fragment ions (*m*/*z*) to those of the corresponding authentic standard compounds, considering the literature data (Table 1 and Table 2). Positive and negative ionization modes were used to identify and quantify the different phenolic compounds of *A. vulneraria* extracts, which were expressed as micrograms per gram of dry weight (µg/g DW).

### 3.5. Spin Trap and EPR Spectroscopy

To determine the EPR scavenging activity of CH_3_O^–^ radicals, a spin-trapping reaction mixture was prepared by adding 300 µL of DMPO (35 mM) to 150 µL of H_2_O_2_ (10 mM) and 150 µL of the extract (or 150 μL of ferulic acid used as reference (0–20 g/L) or 150 μL of pure MeOH used as a control) added in this order. Then, the obtained solutions (~600 µL) were driven inside narrow quartz tubes (2 mm diameter) and introduced into the cavity of the EPR spectrometer, and finally, 150 µL of FeSO_4_ (2 mM) was added [39]. The spectrum was recorded with a spectrometer (Bruker EMX 10/12-Plus, Madrid, Spain) just 10 min after the addition of the FeSO_4_ solution. The following EPR spectrometer settings were applied for all the experiments: microwave power: 20.00 mW; magnetic field amplitude modulation: 1.86 G; frequency modulation: 100 KHz; time constant: 10.24 ms; conversion time: 50 ms; scanning time: 51.20 s; field width: 100 G; gain: 7.10 × 10^3^; and resolution: 1024 points. Ferulic acid and deoxygenated MeOH solutions were used as positive and negative controls, respectively.

### 3.6. In Vitro Antiproliferative Activity by MTT Assay

The ability of *A. vulneraria* extracts to reduce the viability of the three cancer cell lines was determined by the MTT assay according to the method described by Kchaou et al. [40], with some modifications. Three different cell lines (MCF-7, derived from breast adenocarcinoma; HeLa, derived from cervical adenocarcinoma; HepG2, derived from hepatocellular carcinoma) were obtained from ATCC nos. (CCL-2, HB-8065, and HTB-22, respectively). To obtain the appropriate cell concentrations, HeLa, HepG2, and MCF-7 cancer cells were maintained in a 5% CO_2_ incubator at 37 °C, using DMEM medium supplemented with an antibiotic solution of 100 IU/mL penicillin, 100 mg/mL streptomycin, and 10% (*v*/*v*) of heat-inactivated fetal bovine serum [41].

To perform the assay, the cells were seeded in sterile 24-well microplates at a density of 4.7 × 104 cells/well 24 h before the addition of A. vulneraria leaf and flower extracts, both at concentrations of 50 μg/mL and 250 μg/mL. The cells were additionally incubated for 48 h at 37 °C. Then, the cell growth medium was removed, and 120 µL of an MTT reagent (2.5 mg/mL) and 420 µL of sodium succinate (6 g/mL) were added to 1 mL of a fresh culture medium. After 3 h of incubation at 37 °C and 5% CO_2_, the medium was removed, and formazan was resuspended in a DMSO medium containing 0.57% acetic acid and 10% SDS. Negative controls consisted of non-treated cells and the cells treated with a PBS solvent. The absorbance of the different samples was measured at 570 nm using a UV spectrophotometer (Dinko, UV2310 Barcelona, Spain).

### 3.7. Statistical Analysis

Statistical analyses were conducted with the Minitab statistical software (V. 20, München, Germany). The analysis of all the data was carried out in triplicate (*n* = 3), and the results are reported as means ± standard deviations (SDs). Tukey’s test was applied to determine the significant differences among the samples (with α = 0.05).

## 4. Conclusions

This research demonstrated that *A. vulneraria* leaf and flower extracts are composed of different phenolic compounds that could be associated with their potent antiradical and antiproliferative activities. In the leaf extract, 17 phenolic compounds were tentatively identified, among which chlorogenic, caffeic, 2,3-dihydroxybenzoic, p-coumaric, ferulic, sinapinic, 4-hydroxy-2-phenylacetic, and cinnamic acids and kaempferol-3-O-rutinoside flavonoid were the most abundant. By contrast, 15 compounds were identified in flower extract, which was mainly characterized by epicatechin, quercetin, myricetin, delphinidin 3-O sambubioside, rutin, and kaempferol-3-O-rutinoside flavonoids and sinapinic, p-coumaric, and caffeic acids. The high content of flavonoids and phenolic acids in the leaf and flower extracts make *A. vulneraria* an important source of bioactive molecules, which could be used as a functional agent in pharmaceutical products. This study also demonstrated the antiproliferative activity of these extracts (leaf and flower) against the three-cancer cell lines tested (HepG2, HeLa, and MCF-7), although the viability of these cells was determined by the concentration of extract (250 μg/mL > 50 μg/mL), the type of extract (leaf or flower), and the type cancer cell. A deeply powerful approach for the fractionation and identification of the specific phenolic compounds would be proposed in future studies to identify which components, alone or combined, are specifically responsible for the antiproliferative activity of these extracts, along with unraveling of the intracellular mechanisms triggered by these compounds and determining those phenolic compounds that are more potent in scavenging CH_3_O^−^ radicals.

## Figures and Tables

**Table 1:** Identification and quantification of the different compounds present in *A. vulneraria* leaf extract using HPLC-MS.

Peak No.	Tentative Identification	Chemical Formula	RT (min)	Molecular Weight	Ionization Mode	Fragment Ions (*m*/*z*)		Polyphenol Class	Content *	Ref.
						Theoretical (*m*/*z*)	Observed (*m*/*z*)			
L1	Pyrogallol	C_6_H_6_O_3_	6.96	126.1100	[M + H]+	127.0390	127.0391	Other polyphenols	136.94	[20]
L2	Chlorogenic acid	C_16_H_18_O_9_	7.29	354.3087	[M − H]−	353.0878	353.0880	Phenolic acids	1504.62	Std/[21]
L3	4-Hydroxybenzaldehyde	C_7_H_6_O_2_	7.43	122.1213	[M − H]−	121.0295	121.0306	Other polyphenols	1265.74	[20]
L4	Caffeic acid	C_9_H_8_O_4_	8.34	180.1574	[M − H]−	179.0345	179.0350	Phenolic acids	5868.65	Std/[21]
L5	3,4-Dihydroxyphenylglycol	C_8_H_10_O_4_	11.05	170.1626	[M − H]−	169.0506	169.0503	Other polyphenols	57.96	[20]
L6	2,3-Dihydroxybenzoic acid	C_7_H_6_O_4_	11.22	154.1201	[M − H]−	153.0193	153.0203	Phenolic acids	89.73	[20]
L7	p-Coumaric acid	C_9_H_8_O_3_	11.46	164.1580	[M − H]−	163.0401	163.0393	Phenolic acids	106.52	Std/[21]
L8	p-Anisaldehyde	C_8_H_8_O_2_	11.74	136.1479	[M − H]−	135.0451	135.0456	Other polyphenols	2547.22	[20]
L9	Ferulic acid	C_10_H_10_O_4_	13.09	194.1840	[M − H]−	193.0506	193.0502	Phenolic acids	7985.14	Std/[21]
L10	Sinapinic acid	C_11_H_12_O_5_	13.38	224.2100	[M − H]−	223.0612	223.0603	Phenolic acids	3477.81	Std/[22]
L11	4-Hydroxy-2-phenylacetic acid	C_8_H_8_O_3_	14.72	152.1473	[M − H]−	151.0400	151.0408	Phenolic acids	1069.51	[20]
L12	Cinnamic acid	C_9_H_8_O_2_	17.34	148.1586	[M + H]+	149.0597	149.0587	Phenolic acids	7842.12	[20]
L13	2-Hydroxybenzoic acid	C_7_H_6_O_3_	19.02	138.1207	[M − H]−	137.0244	137.0249	Phenolic acids	142.44	[20]
L14	Coumarin	C_9_H_6_O_2_	20.44	146.1427	[M + H]+	147.0441	147.0429	Other polyphenols	651.23	[23]
L15	p-Coumaroyl tartaric acid	C_13_H_12_O_8_	21.88	296.2296	[M − H]−	295.0459	295.0468	Phenolic acids	49.58	[22]
L16	4-Hydroxyphenylpropionic acid	C_9_H_10_O_3_	35.16	166.1739	[M + H]+	165.0557	165.0569	Phenolic acids	133.42	[20]
L17	Kaempferol-3-O-rutinoside	C_15_H_10_O_6_	37.52	286.2363	[M − H]−	285.0404	285.0404	Flavonoids	6314.85	[23]

* Expressed as microgram per g of dry weight (µg/g DW). “Std” indicates identification of components confirmed by a standard. “L” refers to “Leaf”.

**Table 2:** Identification and quantification of the different compounds present in *A. vulneraria* flower extract using HPLC-MS.

Peak No.	Tentative Identification	Chemical Formula	RT (min)	Molecular Weight	Ionization Mode	Fragment Ions (*m*/*z*)		Polyphenols Class	Content *	Ref.
						Theoretical (*m*/*z*)	Observed (*m*/*z*)			
F1	Chlorogenic acid	C_16_H_18_O_9_	7.29	354.3087	[M − H]−	353.0878	353.0880	Phenolic acids	318.55	Std/[21]
F2	Caffeic acid	C_9_H_8_O_4_	8.34	180.1574	[M − H]−	179.0345	179.0350	Phenolic acids	5568.44	Std/[21]
F3	Syringic acid	C_9_H_10_O_5_	8.68	198.1727	[M − H]−	198.05282	197.0453	Phenolic acids	102.209	Std/[24]
F4	(-)-Epicatechin	C_15_H_14_O_6_	10.15	290.2681	[M − H]−	289.0717	289.0717	Flavonoids	1178.12	Std/[22]
F5	p-Coumaric acid	C_9_H_8_O_3_	11.46	164.1580	[M − H]−	163.0401	163.0393	Phenolic acids	5326.11	Std/[21]
F6	Ferulic acid	C_10_H_10_O_4_	13.09	194.1840	[M − H]−	193.0506	193.0502	Phenolic acids	418.63	Std/[21]
F7	Sinapinic acid	C_11_H_12_O_5_	13.38	224.2100	[M − H]−	223.0612	223.0603	Phenolic acids	7699.18	Std/[22]
F8	2,3-Dihydroxybenzoic acid	C_7_H_6_O_4_	13.45	154.1201	[M − H]−	153.0193	153.0203	Phenolic acids	533.36	[22]
F9	Quercetin	C_15_H_10_O_7_	14.50	302.2357	[M + H]+	303.0500	303.0487	Flavonoids	101.41	[22]
F10	Myricetin	C_15_H_10_O_8_	18.27	318.2351	[M + H]+	319.0449	319.0427	Flavonoids	4382.05	Std/[20]
F11	Quercetin	C_15_H_10_O_7_	21.32	302.2357	[M − H]−	301.0354	301.0375	Flavonoids	1154.11	Std/[20]
F12	p-Coumaroyl tartaric acid	C_13_H_12_O_8_	22.17	296.2296	[M − H]−	295.0459	295.0468	Phenolic acids	41.77	[25]
F13	Delphinidin 3-O sambubioside	C_26_H_29_O_16_	25.71	597.4989	[M − H]−	596.1383	596.1363	Flavonoids	64.24	[20]
F14	Rutin	C_27_H_30_O_16_	27.14	610.5175	[M − H]−	609.1525	609.1509	Flavonoids	4982.14	Std/[26]
F15	Kaempferol-3-O-rutinoside	C_15_H_10_O_6_	37.52	286.2363	[M − H]−	285.0404	285.0404	Flavonoids	6314.85	[23]

* Expressed as microgram per g of dry weight (µg/g DW). “Std” indicates identification of components confirmed by a standard. “F” refers to “Flower”.

## Data Availability

Not applicable.

## Sample Availability

Samples of *Anthyllis vulneraria* and the cancer cell lines tested are available from the authors.
